# Temporal cues trick the visual and auditory cortices mimicking spatial cues in blind individuals

**DOI:** 10.1002/hbm.24931

**Published:** 2020-02-12

**Authors:** Monica Gori, Maria Bianca Amadeo, Claudio Campus

**Affiliations:** ^1^ U‐VIP Unit for Visually Impaired People Fondazione Istituto Italiano di Tecnologia Genova Italy

**Keywords:** auditory cortex, blindness, spatial representation, temporal representation, visual cortex

## Abstract

In the absence of vision, spatial representation may be altered. When asked to compare the relative distances between three sounds (i.e., auditory spatial bisection task), blind individuals demonstrate significant deficits and do not show an event‐related potential response mimicking the visual C1 reported in sighted people. However, we have recently demonstrated that the spatial deficit disappears if coherent time and space cues are presented to blind people, suggesting that they may use time information to infer spatial maps. In this study, we examined whether the modification of temporal cues during space evaluation altered the recruitment of the visual and auditory cortices in blind individuals. We demonstrated that the early (50–90 ms) occipital response, mimicking the visual C1, is not elicited by the physical position of the sound, but by its virtual position suggested by its temporal delay. Even more impressively, in the same time window, the auditory cortex also showed this pattern and responded to temporal instead of spatial coordinates.

## 1 INTRODUCTION

Spatial representation is a complex task for the brain (for review Burr & Morrone, 2011; Groh, 2014; Keating & King, 2015). It is mainly resolved by the visual system, which mostly relies on the retinotopic representation of space. The principles that guide the development of spatial representation are still a matter of debate. Blindness is a natural condition through which investigate how space is interpreted, and how the visual cortex is reorganized when visual input is not available.

In young animals, the visual cortex is highly plastic and even retains some plasticity during adulthood (Merabet & Pascual‐Leone, 2010). In blind individuals, this plasticity allows the visual cortex to become colonized by the auditory and somatosensory systems (Sadato et al., 1996; Weeks et al., 2000). In agreement with this idea, functional magnetic resonance imaging (fMRI; Amedi et al., 2007, Lane, Kanjlia, Omaki, & Bedny, 2015, Roder, Stock, Bien, Neville, & Rosler, 2002, Bedny, Pascual‐Leone, Dodell‐Feder, Fedorenko, & Saxe, 2011) and event‐related potential (ERP; Focker, Best, Holig, & Roder, 2012, Kujala et al., 1995) studies show robust and reliable responses to sound alone in the primary visual cortex of blind individuals. In some cases, these neurophysiological results have been associated with enhanced auditory and tactile skills in blind individuals (Fortin et al., 2008; Goldreich & Kanics, 2003; Gougoux et al., 2004; Lessard, Pare, Lepore, & Lassonde, 1998; Roder et al., 1999; Tinti, Adenzato, Tamietto, & Cornoldi, 2006). However, the mere activation of visual cortex, as it has been shown in the cited studies above, is not necessarily indicative of a functional role for these areas. If the lack of vision can drive the functional recruitment of the visual areas and enhancements on the remaining senses, it is possible that the lack of visual input affects the development of some additional processing. For example, disordered auditory spatial maps have been reported in the superior colliculus of owls reared with distorting visual prisms (Knudsen & Knudsen, 1985) and in deprived young ferrets (King & Carlile, 1993). Comparable (but transitory) effects have also been demonstrated in humans (Recanzone, 1998; Zwiers, Van Opstal, & Paige, 2003). Moreover, these neurophysiological results are supported by psychophysical studies showing that visual deprivation impairs some auditory spatial localization skill (Kolarik, Cirstea, & Pardhan, 2013; Kolarik, Pardhan, Cirstea, & Moore, 2017; Wanet & Veraart, 1985). The role of visual recruitment, and the reason why some spatial skills are enhanced for blind individuals yet impaired for others, are still open issues. In 2014, we demonstrated that blind individuals have substantial impairments in performing audio spatial bisection tasks (Gori, Sandini, Martinoli, & Burr, 2014; Vercillo, Burr, & Gori, 2016), in which the participant needs to judge the relative spatial position of the second sound in a sequence of three spatially separated sounds, that is, if the second sound is either closer in space to the first or to the third sound. It is usually an effortless task that sighted children can perform by 6 years of age (Gori, Sandini, & Burr, 2012). We recently reported a possible neural correlate to this behavioral deficit, previously reported through psychophysical methods (Campus, Sandini, Amadeo, & Gori, 2019). Only sighted subjects, who perform the spatial bisection task without difficulty, show an early specific response of the visual cortex during this task (Campus, Sandini, Concetta Morrone, & Gori, 2017). Specifically, in sighted and not in blind participants a component has been observed between 50 and 90 ms after the second sound in the spatial bisection task, which represents a crucial time window in the earliest stages of sensory processing. The early occipital response described in sighted people was strong and contralateral to the spatial position of the sound, mimicking many characteristics of the C1 ERP component usually elicited by visual stimuli (Di Russo, Martinez, Sereno, Pitzalis, & Hillyard, 2002). Interestingly, the same contralateral occipital activation has not been recorded in blind individuals after the same acoustic stimulation (Campus et al., 2019) and in late blind people who lost their vision more than 20 years ago (Amadeo, Campus, & Gori, 2019). These results suggest that early blindness affects strategies and neural circuits underlying the construction of sophisticated spatial metrics through long‐term neural plasticity.

Although the bisection task requires complex attentional and memory skills, it is hard for blind individuals only in the spatial domain. When they perform a temporal bisection task, which involves the evaluation of the temporal intervals between three sounds, their performance is as good as those of sighted individuals (Gori et al., 2014), and their cortical activations are similar (Campus et al., 2017; Campus et al., 2019). We recently showed that temporal information could be used by blind individuals to infer spatial coordinates during the spatial bisection task (Gori, Amadeo, & Campus, 2018). Indeed, we observed that the deficit in spatial bisection disappears if blind individuals are presented with coherent temporal and spatial cues, and increases when spatial and temporal information is provided in conflict, suggesting that spatial representation in blind individuals is strongly influenced by temporal representation.

Why do blind individuals use temporal cues to solve this spatial task? Almost 100 years ago, Jean Piaget and Inhelder (1962) stated that the temporal metric is strictly related to spatial metric development. “Space is a still of time, while time is space in motion” (Piaget, 1927, p. 2). Jean Piaget did not, however, discuss the role of different sensory modalities on this link. Our results (Gori et al., 2018) suggest that the visual experience plays a crucial role in learning to decode complex spatial coordinates: without visual experience, temporal metrics influence spatial metrics. In this work, we hypothesized that if blind individuals use temporal cues to represent space, their visual and possibly auditory cortices should also respond to temporal instead of spatial auditory coordinates.

To test this hypothesis, EEG and behavioral responses were recorded while blind people performed spatial and temporal bisection tasks, in which coherent and conflicting spatiotemporal information was presented. As predicted, we demonstrated that when coherent spatiotemporal cues were presented (e.g., short space associated with short time) to blind individuals, the same early contralateral occipital response emerged as that which is observed in sighted individuals. This suggests that the same circuits that are activated by spatial cues in sighted individuals, could be activated by temporal cues in blind individuals. Moreover, we observed that when conflicting spatiotemporal information was presented (e.g., short space associated with a long time), the early occipital component remained always contralateral to the spatial position of the second sound in space for sighted individuals. For blind individuals it was inverted and was based on the virtual position of the second stimulus defined by its temporal delay. More interestingly, we observed that, in the conflicting conditions, the auditory cortex of blind individuals was also activated contralaterally to the position of the second sound suggested by its temporal delay, while the same was not true for sighted individuals. These results suggest that the temporal cue results in a strong illusion of sounds being perceived as coming from a different position than they really are and, surprisingly, tricks the earliest stages of both visual and auditory processing. Thus, cortical reorganization in blind individuals does not always seem to be adaptive and, in some cases, drives a spatial misperception that can affect capabilities for interacting with the environment.

## 2 RESULTS

**Figure 1:**
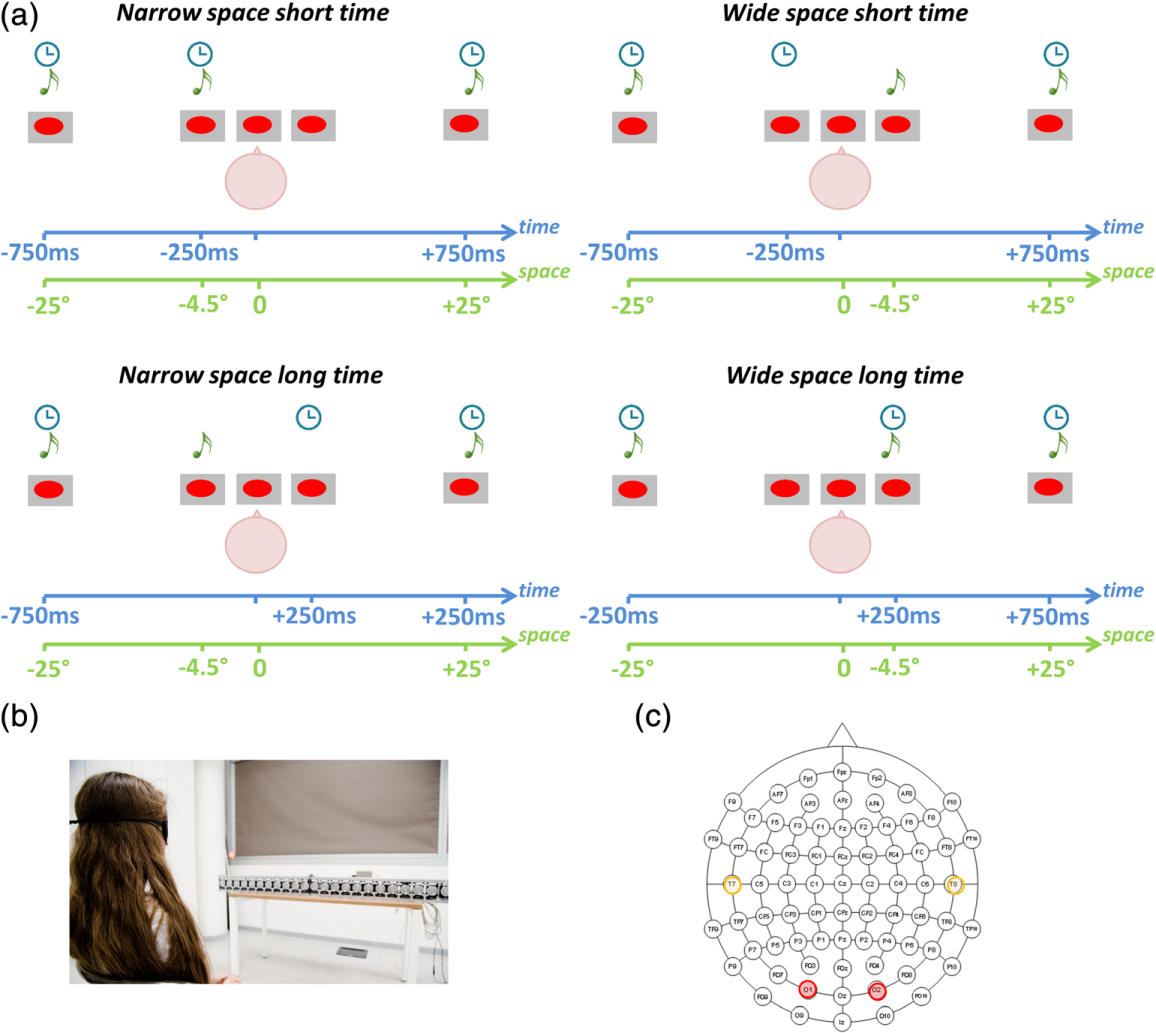
Spatial and temporal audio bisection tasks. (a) Experimental design: participants listened to three sounds delivered at three different spatial positions and times. Subjects judged the relative spatial (i.e., spatial bisection) or temporal (i.e., temporal bisection) position of S2 with respect to S1 and S3. S1 and S3 were always delivered from −25° and +25°, respectively (with 0° representing the central speaker, negative values on the left and positive values on the right), and at −750 and + 750 ms, respectively (with 0 ms representing the halfway point of the trial duration). S2 occurred randomly and independently from ±4.5° in space and at ±250 ms. Interaction of spatial distance and temporal delay between sounds gave rise to four different conditions: *narrowSpace_shortTime* (i.e., S2 from −4.50° at −250 ms, see A Top Left), *narrowSpace_longTime* (i.e., S2 from −4.50° at +250 ms, see A Top Right), *wideSpace_longTime* (i.e., S2 from +4.50° at +250 ms, see A Bottom Left), *wideSpace_shortTime* (i.e., S2 from +4.50° at −250 ms, see A Bottom Right). Narrow/wide first distances correspond to S2 delivered from the left (−4.5°) or right (+4.5°) side of the subject, respectively. (b) Experimental setup. Subjects were blindfolded and sounds were delivered using free‐field speakers placed in the lower visual hemifield. (c) Electrode montage for EEG recording and electrodes considered in EEG data analysis. In orange, left (T7) and right (T8) temporal electrodes; in red, left (O1) and right (O2) occipital electrodes

Sixteen blindfolded sighted individuals and 16 early blind subjects performed a spatial and a temporal auditory bisection task on a sequence of three sounds. The middle sound could be randomly and independently delivered at two different spatial positions and according to two different temporal lags, giving rise to coherent (*narrowSpace_shortTime*, *wideSpace_longTime*) and incoherent (*narrowSpace_longTime*, *wideSpace_shortTime*) spatiotemporal conditions (see Figure 1). Subjects were asked whether the first distance/interval (between the first and the second sound) was temporally longer (temporal bisection), or whether it was spatially larger (spatial bisection) than the second distance/interval (between the second and the third sound). Narrow (i.e., narrowSpace) and wide (i.e., wideSpace) first spatial distances corresponded to the second sound delivered from the left (−4.5°) or right (+4.5°) side of the subject, respectively. Both psychophysical responses and ERPs were collected. Our results revealed differences between sighted and blind subjects in the conflicting conditions for spatial but not temporal bisection tasks. The effect was evident in both the psychophysical and EEG results, suggesting that temporal cues alter the recruitment of the visual and auditory cortices during auditory spatial representation in blindness.

**Figure 2:**
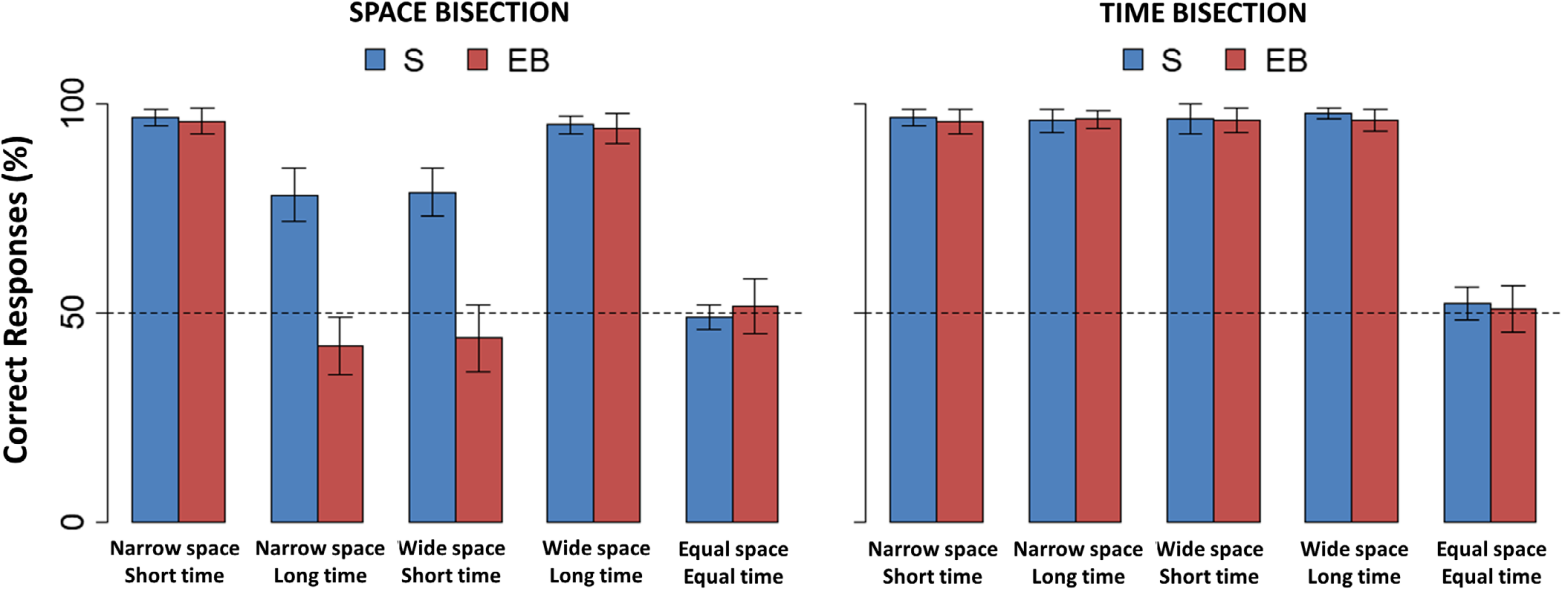
Behavioral results for the spatial (left) and temporal (right) bisection tasks. Percentage of correct responses (mean ± *SEM*) is reported for sighted (S, blue) and early blind (EB, red) individuals. Specifically, performance is shown for coherent conditions (i.e., *narrowSpace_shortTime*, *wideSpace_longTime*), conflicting conditions (i.e., *narrowSpace_longTime*, *wideSpace_shortTime*), and catch trials (i.e., *equalSpace_equalTime)*. Dashed lines represent chance level (i.e., 50% of correct answer)

An ANOVA was performed to examine the differences in spatial bisection performance and demonstrated a significant interaction (*F*[3,90] = 13.03, *p* < .0001, generalized eta squared *η*_g_^2^ = 0.16) between GROUP and CONDITION. Although there was a slight decrease in performance, sighted participants succeeded in the spatial bisection task independently of the spatiotemporal coherence or incoherence (Figure 2, left). During coherent conditions, both groups showed a similar high percentage of correct responses (for *narrowSpace_shortTime*: *t*(30) = 0.22, *p* = .82; for *wideSpace_longTime*: *t*(30) = 0.20, *p* = .84). However, in the conflicting conditions, blind participants showed a significant deficit in performance when compared to sighted participants (for *narrowSpace_longTime*: *t*(30) = 3.9, *p* = .002; *wideSpace_shortTime*: *t*(30) = 3.52, *p* = .005). Indeed, while sighted people always performed above chance level in the conflicting conditions, blind participants systematically performed below chance level. However, the spatiotemporal coherence or incoherence only produced a different response in blind people for the question related to space. No significant interaction (*F*[3,90] = 1.34, *p* = .27, *η*_g_^2^ = 0.03) appeared from the ANOVA involving temporal bisection performance (Figure 2, right).

**Figure 3:**
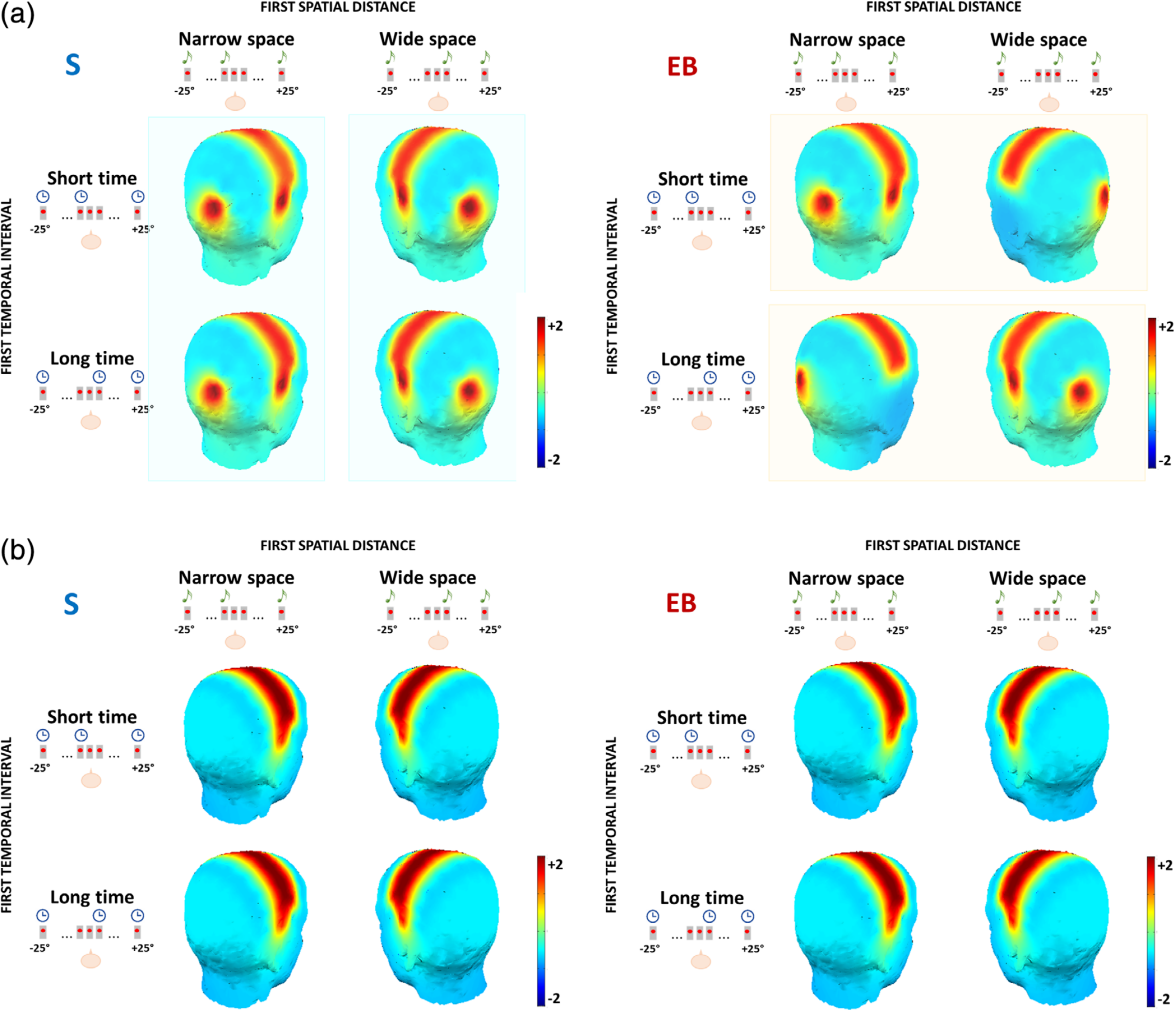
Scalp maps of the mean ERP amplitude in the selected (50–90 ms) time window after S2 of the spatial (a) and temporal (b) bisection tasks, for sighted (S, left) and early blind (EB, right) individuals. The first spatial distance could be either narrow (i.e., S2 delivered from −4.5°) or wide (i.e., S2 delivered from +4.5°), and the first temporal interval could be either short (i.e., S2 delivered at −250 ms) or long (i.e., S2 delivered at +250 ms). For sighted subjects, the contralateral occipital and temporal activation in the spatial bisection (a) depends on the first spatial distance. For blind subjects, the same contralateral occipital and temporal activation depends on the first temporal interval

During the EEG analyses, typical auditory processing was observed in central electrodes for sighted and blind individuals during both spatial and temporal bisection tasks (Figure S1). Based on our hypothesis, we subsequently focused our analyses on occipital and temporal activation 50–90 ms after S2 of the spatial bisection task, using the temporal bisection task as a control. In Figure 3, we report the scalp maps of the mean amplitude in the 50–90 ms time window, in response to S2 of the spatial (Figure 3a) and temporal (Figure 3b) bisection task for sighted (left) and blind (right) participants. We observed several main differences on the cortical response between groups during the spatial (Figure 3a) but not the temporal (Figure 3b) bisection task. For the coherent spatiotemporal conditions of the space bisection task (*narrowSpace_shortTime*, *wideSpace_longTime*), the physical position of S2 elicited a specific occipital and temporal contralateral response both in sighted (Figure 3, left, in agreement with Campus et al., 2017) and in blind subjects (Figure 3a, right). However, for the conflicting spatiotemporal stimuli in the spatial bisection task (i.e., *narrowSpace_longTime*, *wideSpace_shortTime*) a different pattern emerged: the physical position of S2 still elicited a specific occipital and contralateral temporal response in sighted subjects (Figure 3a, left), while the response of blind subjects was inverted and ipsilateral (Figure 3a, right). The occipital and temporal sites of sighted participants showed the same response observed in the coherent conditions, contralateral with respect to the second sound position in space. The response of blind participants in the same time window, however, was strongly ipsilateral to the spatial position of S2, as if the subjects were using the virtual position of the sound determined by its temporal delay.

As suggested by the scalp maps, the ANOVA on the mean occipital ERP amplitudes in the selected time window confirmed an interaction (*F*[3,90] = 47.05, *p* < .0001, *η*_g_^2^ = 0.27) between HEMISPHERE, CONDITION, and GROUP for the spatial bisection task. As expected, each HEMISPHERE showed an interaction CONDITIONxGROUP (*left*: *F*(3,90) = 15.64, *p* < .0001, *η*_g_^2^ = 0.28; *right*: *F*(3,90) = 14.41, *p* < .0001, *η*_g_^2^ = 0.26).

**Figure 4:**
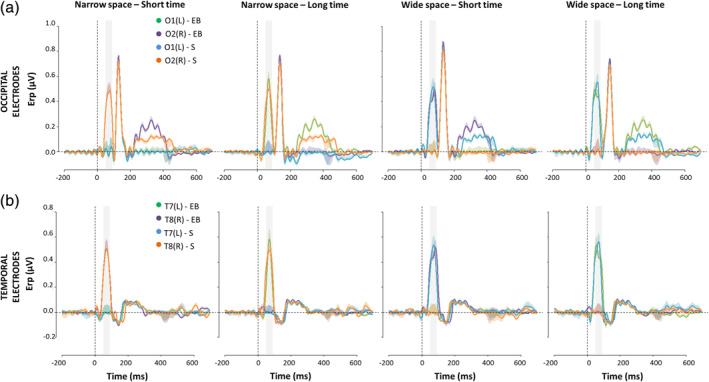
ERPs (mean ± *SEM*) elicited by S2 during spatial bisection task in occipital (a) and temporal (b) electrodes. Both left (O1, T7) and right (O2, T8) electrodes are reported for sighted (S) and early blind subjects (EB), considering each coherent (i.e., *narrowSpace_shortTime*, *wideSpace_longTime*) and conflicting (i.e., *narrowSpace_longTime*, *wideSpace_shortTime*) condition. For *narrowSpace_shortTime* and *narrowSpace_longTime* conditions, O1 and T7 are ipsilateral while O2 and T8 are contralateral with respect to the physical location of the stimulus. For *wideSpace_longTime* and *wideSpace_shortTime* conditions, O1 and T7 are contralateral while O2 and T8 are ipsilateral with respect to the physical location of the stimulus. On the *x*‐axis, *t* = 0 is sound onset. The shaded area delimits the selected time window (50–90 ms)

**Figure 5:**
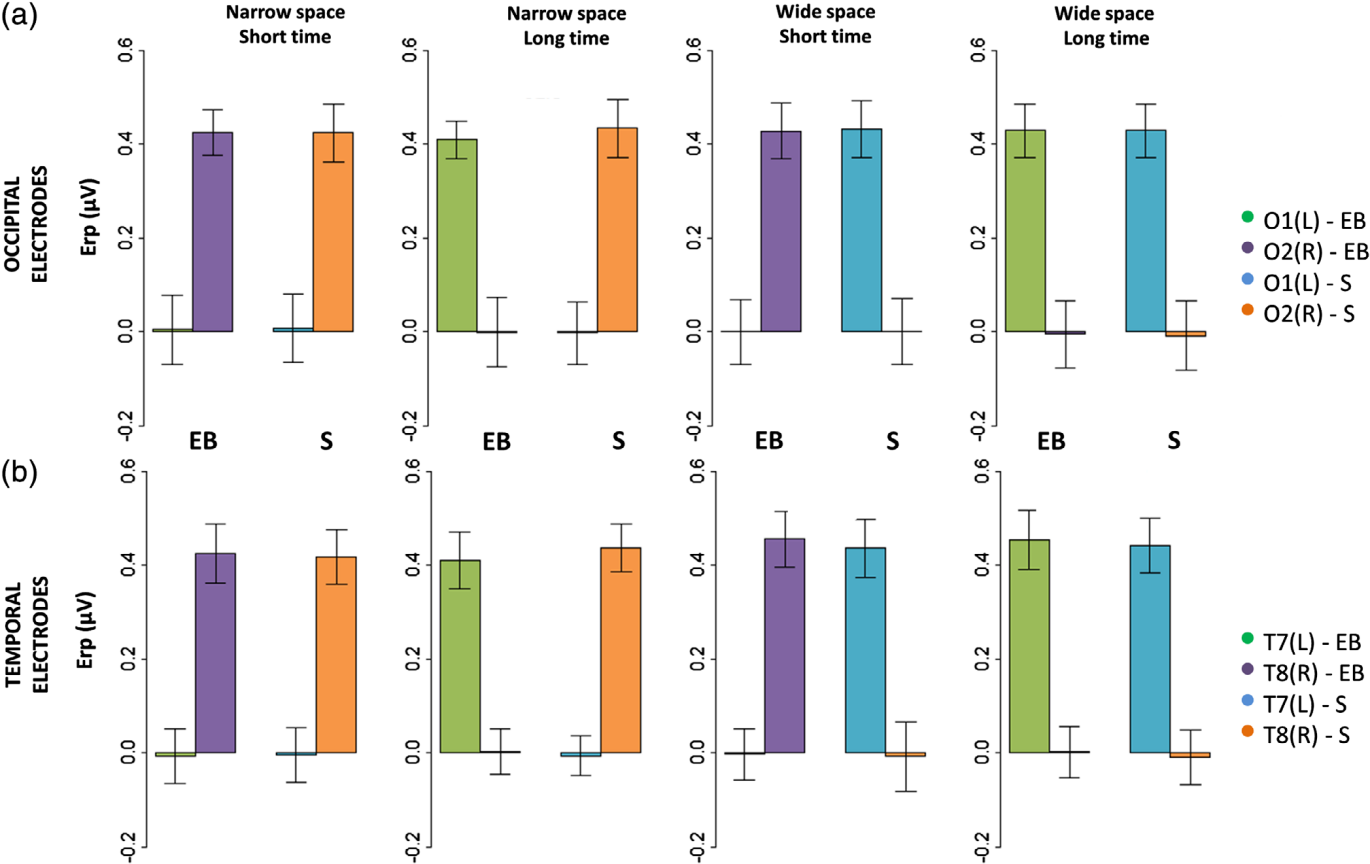
Mean (±*SEM*) ERP amplitude in the selected (50–90 ms) time window after S2 of the spatial bisection task in occipital (a) and temporal (b) electrodes. Both left (L: O1, T7) and right (R: O2, T8) electrodes are reported for sighted (S) and early blind subjects (EB), considering each coherent (i.e., *narrowSpace_shortTime*, *wideSpace_longTime*) and conflicting (i.e., *narrowSpace_longTime*, *wideSpace_shortTime*) condition

In the sighted group (Figure 4a and Figure 5a), the early occipital response to S2 was highly significant and lateralized with respect to S2 phisical spatial position for both coherent and conflicting conditions (Table 1a). The left occipital electrode showed a response only when the sound was physically placed on the right side of the subject (i.e., *wideSpace_shortTime*, *wideSpace_longTime*) but not when it was on the left (i.e., *narrowSpace_shortTime*, *narrowSpace_longTime*). Symmetrically, the right occipital electrode showed a response only when S2 was delivered from the left, but not when it was delivered from the right. For the blind group (Table 1b) the same pattern was evident only for the coherent conditions. Indeed, in the coherent conditions, when compared with sighted people, the left occipital electrode of blind individuals showed a similarly strong response when the sound was delivered from the right (*wideSpace_longTime*) and a similarly weak response when it was delivered from the left (*narrowSpace_shortTime*). Symmetrically, the right occipital electrode showed a similarly strong response when S2 was delivered from the left (*narrowSpace_shortTime*), and a similarly weak response when it was delivered from the right (*wideSpace_longTime*). However, during conflicting conditions, blind subjects showed topographically reversed responses compared to that of sighted subjects: the response seemed to be contralateral to the virtual position of the stimulus, determined by its temporal delay and not by its actual spatial location. In the conflicting conditions, when compared with sighted subjects, the left occipital electrode of blind subjects showed a much stronger response when S2 was temporally closer to S3 (right) but physically delivered from the left of the subject (*narrowSpace_longTime*). Conversely, the response of blind subjects was much weaker when the sound was temporally closer to S1 (left) but physically delivered from the right (*wideSpace_shortTime*). Symmetrically, the right occipital electrode showed a much stronger response when S2 was temporally closer to S1 but physically delivered from the right side of the subject (*wideSpace_shortTime*), while a much weaker response was elicited when the sound was temporally closer to S3 but physically delivered from the left side (*narrowSpace_longTime*). These results suggest that blind subjects were processing the spatial signals based on temporal properties.

**Table 1:** Results of post hoc comparisons for the spatial bisection task

*(a) Sighted group*				
Condition	Area	Hemisphere	*t*(15)	*p*
*narrowSpace_shortTime*	Occipital	Right	6.89	<.0001*
		Left	0.12	.91
	Temporal	Right	7.17	<.0001*
		Left	−0.06	.96
*narrowSpace_longTime*	Occipital	Right	7.01	<.0001*
		Left	−0.03	.97
	Temporal	Right	8.51	<.0001*
		Left	−0.13	.90
*wideSpace_shortTime*	Occipital	Right	0.005	.99
		Left	7.64	<.0001*
	Temporal	Right	−0.10	.92
		Left	7.11	<.0001*
*wideSpace_longTime*	Occipital	Right	−0.11	.91
		Left	7.23	<.0001*
	Temporal	Right	−0.16	.86
		Left	7.60	<.0001*

*(b) Sighted group vs. blind group*				
Condition	Area	Hemisphere	*t*(30)	*p*
*narrowSpace_shortTime*	Occipital	Right	0.002	.99
		Left	−0.03	.97
	Temporal	Right	0.07	.94
		Left	−0.05	.96
*narrowSpace_longTime*	Occipital	Right	−4.51	.0004*
		Left	5.30	<.0001*
	Temporal	Right	−6.13	<.0001*
		Left	5.65	<.0001*
*wideSpace_shortTime*	Occipital	Right	4.61	.0002*
		Left	−4.77	.0002*
	Temporal	Right	4.88	.0001*
		Left	−5.36	<.0001*
*wideSpace_longTime*	Occipital	Right	0.03	.97
		Left	0.003	.98
	Temporal	Right	0.15	.87
		Left	0.15	.88

Surprisingly, an ANOVA of the mean temporal ERP amplitude in the same time window added additional important evidence: the same pattern characterizing the occipital sites involved temporal sites (Figure 4b and Figure 5b). Statistical analyses revealed not only a significant interaction HEMISPHERExCONDITIONxGROUP (*F*(3,90) = 45.06, *p* < .0001, *η*_g_^2^ = 0.32), but also similar results from the follow‐up ANOVAs and posthoc *t*‐tests. For each HEMISPHERE, we found a significant interaction CONDITIONxGROUP (*left*: *F*(3,90) = 14.50, *p* < .0001, *η*_g_^2^ = 0.31, *right*: *F*(3,90) = 20.14, *p* < .0001, *η*_g_^2^ = 0.33). As expected for typical auditory processing (Naatanen & Picton, 1987), the activation in temporal electrodes for sighted people was determined by the spatial position of S2 in all conditions, independently of the temporal delays of the stimulus (Table 1a). For blind participants, however, time cues attracted the neural temporal response in the early time window: during the conflicting conditions, the response in temporal electrodes was ipsilateral to the stimulus location in space and contralateral to its virtual position determined by temporal coordinates. When comparing the response of blind with that of sighted subjects, similar activations were observed for the coherent but not for the conflicting conditions (Table 1b).

In support of the specificity of temporal attraction of space in blindness, the ANOVA did not reveal any significant interaction considering CONDITIONxHEMISPHERExGROUP for the temporal bisection task, nor for the temporal (*F*(3,90) = 0.65, *p* = .58, *η*_g_^2^ = 0.006), nor for the occipital (*F*(3,90) = 1.29, *p* = .28, *η*_g_^2^ = 0.009) areas. During temporal bisection, when subjects were asked to evaluate timing presentations of sounds, neither sighted nor blind subjects showed occipital amplification and both groups were unaffected by the cross‐domain conflict (see Figure S2).

When focusing on occipital electrodes (Figure 4a), we observed a gain modulation of later responses (P140) for both sighted and blind subjects independently of the condition. In line with previous studies (Campus et al., 2017; Campus et al., 2019), this amplification was selective for S2 during the spatial task and did not involve a lateralization effect. The selected time window was the first to show a modulation due to the task, while a later modulation seems to occur, likely involving the auditory‐evoked contralateral occipital activation, previously observed in sighted individuals between 250 and 400 ms (McDonald, Stormer, Martinez, Feng, & Hillyard, 2013). We previously reported that this later contralateral occipital ERP response was lower and not lateralized during a spatial bisection task for blind participants (Campus et al., 2019). Interestingly, by manipulating both spatial and temporal information, we observed that this later response was lower and not lateralized during spatial bisection because it is actually more sensitive to the temporal cues of the stimuli. Indeed, its topography in blind subjects resembled that of the early response (contralateral to the virtual and not real position of S2 in the conflicting conditions), and was even more pronounced.

**Figure 6:**
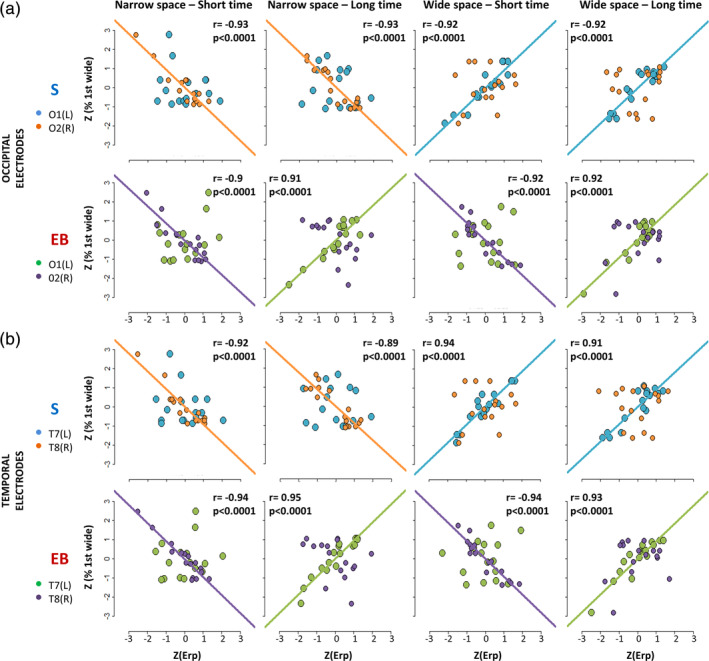
Correlations between the perceived localization of S2 during the spatial bisection task and mean ERP amplitude in occipital (a) and temporal (b) electrodes within the 50–90 ms window, evaluated for sighted (S) and early blind (EB) groups separately. For each subject and condition (from left to right: *narrowSpace_shortTime, narrowSpace_longTime, wideSpace_shortTime, wideSpace_longTime*), individual *z*‐transformed ERP amplitude in O1/T7 (L = left hemisphere; blue for sighted, green for blind) and O2/T8 (R = right hemisphere; orange for sighted, violet for blind) is plotted against the *z*‐transformed percentage of trials in which the subject perceived the first distance as wider (i.e., perceiving S2 delivered from the right). Regression lines represent significant correlations, with corresponding Pearson correlation coefficient (*r*) and significant level (*p*). The color of lines is the same as the points showing significant correlations

To probe whether the response modulation is related to the perceived rather than the physical position of S2, we correlated individual ERP responses recorded in occipital and temporal electrodes for each condition of the spatial bisection task with the percentage of trials in which the subject perceived the first distance as wider (S2 perceived as delivered from the right; see Section 4 for more details). As evident in Figure 6, the ERP amplitude in O1/O2 (Figure 6a) and T7/T8 (Figure 6b) was significantly associated with the performance for both sighted and blind subjects, but the correlational patterns varied across conditions and groups.

For sighted subjects, the percentage of trials in which participants reported the first distance as wider negatively correlates with the ERP amplitude in O2 and T8 when the first distance was narrow, meaning S2 was delivered from the left (*narrowSpace_shortTime*, O2: *r* = −.93, *p* < .0001, T8: *r* = −.92, *p* < .0001, *narrowSpace_longTime*, O2: *r* = −.93, *p* < .0001, T8: *r* = −.89, *p* < .0001), and positively correlates with the ERP amplitude in O1 and T7 when the first distance was wide, meaning S2 delivered from the right (*wideSpace_shortTime*, O1: *r* = .92, *p* < .0001, T7: *r* = .94, *p* < .0001, *wideSpace_longTime*, O1: *r* = .92, *p* < .0001, T7: *r* = .91, *p* < .0001). Hence, for sighted people, the association between the perceived position of S2 and contralateral occipital and temporal ERP amplitude existed independently of the temporal coordinates of the stimulus. For blind participants, we can observe similar results for the coherent conditions but not for the conflicting conditions. Indeed, as with sighted individuals, there was a negative correlation between the percentage of trials in which participants reported the first distance as wider and ERP amplitude in O2 (*r* = −.9, *p* < .0001) and T8 (*r* = −.94, *p* < .0001) for the condition *narrowSpace_shortTime*, and a positive correlation between the percentage of trials in which participants reported the first distance as wider and ERP amplitude in O1 (*r* = .92, *p* < .0001) and T7 (*r* = .93, *p* < .0001) for the condition *wideSpace_longTime*. Contrarily, for the conflicting conditions, the correlation between early ERP amplitude in O1/O2 and T7/T8 and individual performance was very strong for blind subjects but inverted in lateralization and slope. Although S2 was delivered from the left in the *narrowSpace_longTime* condition, there was a positive correlation between perceiving the first distance as wider and the ERP amplitude in O1 (*r* = 0.91, *p* < .0001) and T7 (*r* = 0.95, *p* < .0001). Accordingly, although S2 was delivered from right in the *wideSpace_shortTime* condition, perceiving the first distance as wider negatively correlated with the ERP amplitude in O2 (*r* = −0.92, *p* < .0001) and T8 (*r* = −0.94, *p* < .0001). Thus, the lateralization of the observed occipital and temporal responses was closely related to the perceived more than physical spatial localization of the stimulus. For sighted individuals, the perceived position almost always coincided with the physical one, whereas for blind individuals, the perceived position was influenced by the temporal delay of the stimuli. This supports the hypothesis that blind individuals use temporal cues to determine the spatial position of sound in space.

We can exclude the idea that the effect originated from spurious eye‐movement toward the apparent location of the sound. Average response of eye deviation measured by EOG was not significantly different from zero when grouping responses by the position of S2, for both physical (as for the lowest P value, *Sighted*: *t*(15) = 0.62, *p* = .54, *Blind*: *t*(15) = 0.82, *p* = .42) and perceived intervals (as for the lowest P value, *Sighted*: *t*(15) = 0.72, *p* = .48, *Blind*: *t*(15) = 1.11, *p* = .29).

**Figure 7:**
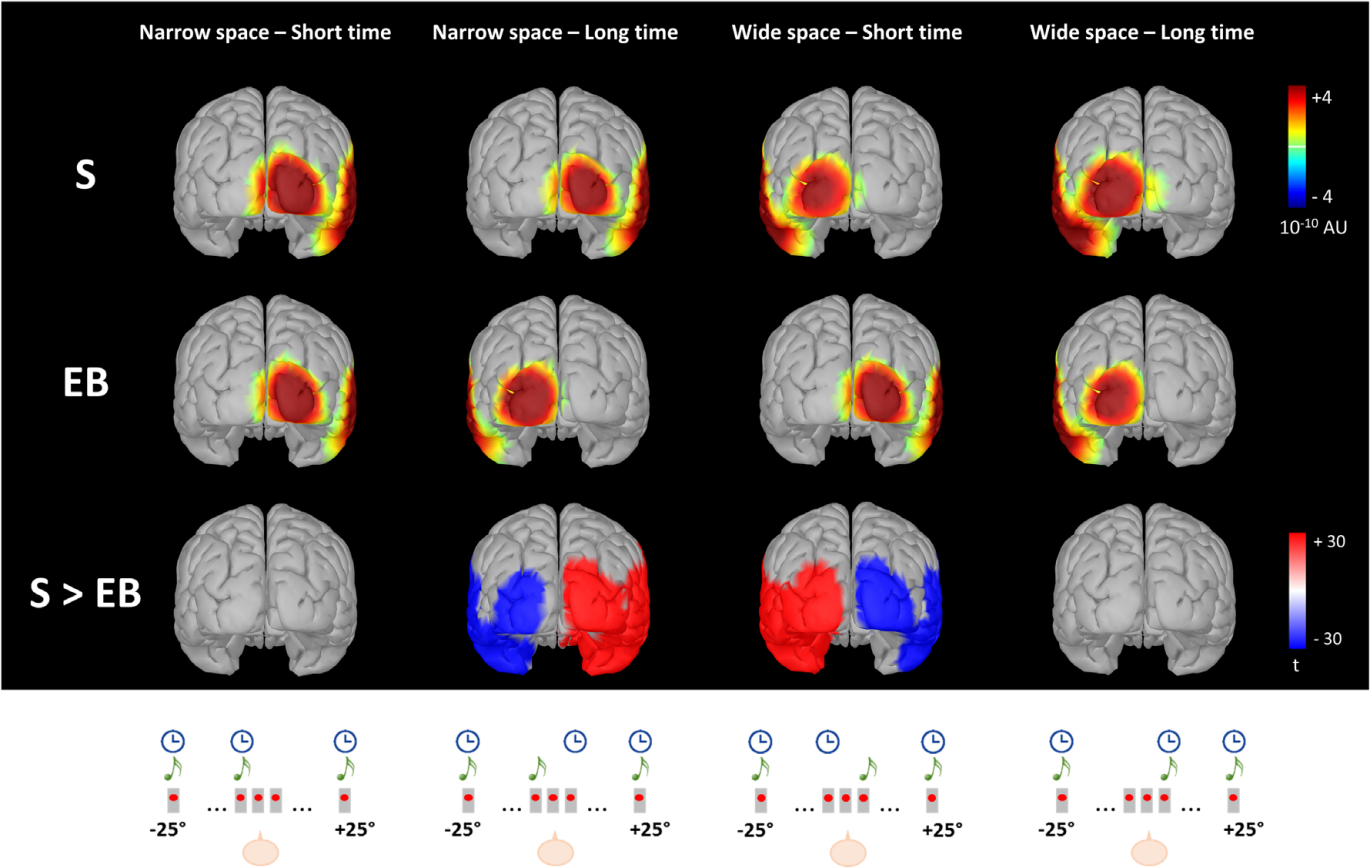
Average source activity elicited by S2 of the spatial bisection task within the selected (50–90 ms) time window is compared between sighted (S) and early blind (EB) individuals across conditions. From left to right: *narrowSpace_shortTime, narrowSpace_longTime, wideSpace_shortTime, wideSpace_longTime*. The first two lines represent average normalized source activation for sighted (first row) and blind (second row) subjects in arbitrary (normalized) units (AU). No masks have been applied. The last line reports the results of paired two‐tailed *t*‐tests; the scale is in terms of *t*‐statistic. Significant values of *t*‐statistic are displayed: reddish and bluish colors indicate stronger activations in sighted and blind subjects, respectively, while the intensity indicates the magnitude of t (i.e., the significance of the difference). Only *t* values corresponding to *p* < .0001 after FDR correction are displayed. At the bottom, a schematic representation of each condition is displayed (see Figure 1)

To test that the occipital and temporal differences between sighted and blind participants actually involved generators in the visual and auditory cortices, respectively, we compared the two groups at source level, considering each coherent and conflicting condition separately. As expected, we did observe significant differences between sighted and blind people for the conflicting conditions (Figure 7).

Sighted subjects always showed a stronger occipital and temporal activation contralateral to the spatial position of the sound, whereas blind subjects during the conflicting conditions exhibited activation in occipital and temporal cortical areas, contralateral to the sound virtual position determined by the temporal information. Specifically, an involvement of left occipital and temporal areas appeared when the stimulus was played from the left but temporally closer to S3 (*narrowSpace_longTime*), and an involvement of right occipital and temporal areas appeared when the stimulus was played from the right but temporally closer to S1 (*wideSpace_shortTime*). Differences between groups were not present for coherent conditions.

## 3 DISCUSSION

In this work, we demonstrated that the neural correlates of deficits previously reported in blind individuals for spatial bisection (Gori et al., 2014), reflect a different organization of the occipital and auditory cortex, based on the use of temporal rather than spatial cues to represent space. We recently reported that sighted but not blind individuals showed an early occipital response, which mimics many features of the C1 ERP observed in visual tasks, during an audio spatial bisection task (Campus et al., 2017; Campus et al., 2019). Here we showed that the different activation in blind individuals is due to alternative processing of the spatial stimuli based on their temporal properties. These results suggest that lack of vision could result in the development of a new strategy to represent space where the occipital and auditory circuits use temporal estimations to interpret spatial metrics.

To distinguish between spatial and temporal information we used a spatial bisection task, in which temporal cues can be manipulated independently to spatial cues and spatial and temporal signals can be congruently or incongruently presented. We replicated the spatial bisection task but presented conflicting and nonconflicting spatiotemporal information through the manipulation of the second stimulus, while behavioral and EEG data were collected. In two conditions, spatial distances and temporal intervals between the sounds were coherent: a wide spatial distance was associated with a long temporal interval, and a narrow spatial distance with a short temporal interval. In the other two conditions, the spatial distances and temporal intervals were incongruent: a wide spatial distance was associated with a short temporal interval, and a narrow spatial distance was associated with a long temporal interval. Sighted individuals succeeded in all tasks and showed lateralized occipital activation of the visual cortex opposite to the actual spatial position of the second sound. In the coherent spatiotemporal conditions, blind individuals also succeeded and showed a cortical activation similar to sighted individuals, contralateral to the spatial and temporal position of the second stimulus. Thus, by presenting coherent spatiotemporal information we observed an early occipital response in both groups. For the first time, blind people displayed the lateralized activation which was not evident in previous work where the temporal factor was not taken into account (Campus et al., 2019). Since no activation was evident for the blind group when only spatial information was provided, without the help of temporal cues (Campus et al., 2019), one may consider the activation observed here to be associated with the introduction of the temporal cue (coherent with the spatial one). However, the coherent conditions did not allow for disentangling between the role of the temporal and spatial cues on the cortical response. To examine this point further we designed a conflicting condition where spatial and temporal information did not coincide. If the cortical activation observed in blind individuals was due to the presence of temporal information associated with spatial information, then we should observe a different pattern of responses between sighted and blind subjects for this condition. Sighted individuals demonstrated the expected pattern, with lateralized occipital activation of the visual cortex opposite to the actual spatial position of the stimulus. Contrarily, the occipital cortex of blind participants showed lateralized activation that aligned with the “temporal” position of the stimulus. Left occipital activation was present for longer temporal intervals and narrower spatial distances (second sound delivered from the left), while right occipital activation for shorter temporal intervals and wider spatial distance distances (second sound delivered from the right). Moreover, when we correlated the individual sound localization performance (i.e., reporting a wider first distance which means perceiving the second sound closer to the last one) with the mean amplitude of ERP response in the early time window, we observed a strong correlation for both sighted and blind groups. These correlations highlight that the spatial selectivity of the observed early occipital response is closely linked to the perceived spatial localization of the stimulus, which almost always coincides with the physical position for sighted but not for blind individuals. Indeed, for blind individuals, the laterality of the ERP component reflects the perceived localization which is determined by the temporal delay of the stimulus and not by its physical position.

Even more interestingly, the same pattern of responding characterized the auditory cortices of blind individuals. Indeed, during the conflicting conditions, the temporal sites of blind participants were activated contralaterally to the position of the second sound suggested by its temporal delay instead of spatial coordinates. Accordingly, the activation in temporal scalp sites correlates with performance. Thus, the temporal cue not only triggered a misperception of the stimulus in the occipital cortex but also an auditory illusion which tricked the early stages of auditory processing. Considering these results, we can speculate that lack of vision might result in the construction of multi‐sensory spatial maps projected onto the retinotopic maps of the visual cortex, based on temporal instead of spatial coordinates. This occipital reorganization may also lead to cortical reorganization in auditory areas. This hypothesis is in line with other studies demonstrating that neural changes following blindness also affects nonvisual areas, such as the auditory (e.g., Elbert et al., 2002; Korte & Rauschecker, 1993) and the somatosensory (e.g., Park et al., 2009; Sterr et al., 1998) cortices.

This work is not the first study reporting the activation of early sensory cortices reflecting perceptual rather than physical characteristics of the stimulus. For instance, in sighted people, the brain exhibits sensitivity to the presence of illusory contour stimuli as early as 90 ms poststimulus onset (Murray et al., 2002), and its response when falsely perceiving the presence of a stimulus (when the stimulus is physically absent) is similar to when it correctly detects its physical presence (Ress & Heeger, 2003). In further support of the argument that brain activity can be more closely related to perceptual rather than physical properties of the stimulation, visual‐evoked potentials are sensitive at early latency to the beep‐flash illusion (Shams, Kamitani, Thompson, & Shimojo, 2001; Watkins, Shams, Tanaka, Haynes, & Rees, 2006), in which the presence of irrelevant sounds modifies the perception of a simple visual stimulus (Shams, Kamitani, & Shimojo, 2000).

The present study provides neurophysiological evidence for the temporal attraction of space reported in blind people with psychophysical methods (Gori et al., 2018). It is the first study to report that early occipital activation to sound is modulated by the temporal properties of the stimulus in blind individuals. This early response to sound shows generators in the visual cortex (Campus et al., 2017), as different from the N48 previously described by others in the parietal areas (Schroeder, Molhom, Lakatos, Ritter, & Foxe, 2004). This is also the first study to report that early auditory activation to sound in blind individuals (i.e., N1a; Naatanen & Picton, 1987) is modulated by temporal as opposed to spatial coordinates of the stimulus. We can exclude the argument that our effects merely originate from different attentional skills to left or right positions of sounds, or different attentional skills between sighted and blind individuals. Although we found a contralateral activation of temporal cortices, as expected for the processing of auditory stimuli (Campus et al., 2017; Naatanen & Picton, 1987), we did not observe a contralateral modulation in central electrodes, which are those usually showing strong attentional responses to sounds, for either sighted or blind individuals (Lange, Kramer, & Roder, 2006; Roder et al., 1999). Similarly, attention to space can be expected to weakly affect early ERPs, such as the observed occipital response and the N1a (Lange et al., 2006, Roder et al., 1999). Moreover, when the temporal bisection task was performed as a control, the responses were similar across all individuals and conditions, suggesting that the cross‐domain illusion was specific for the spatial task. For the blind group, the activation in temporal sites was also always contralateral to the stimulus position in space during temporal bisection, independently of conditions. This result shows that when subjects need to build temporal but not spatial metrics, the auditory cortices are able to represent space in a spatially selective manner, confirming the specific modulation of the spatial task.

Previous work showed that vision is the sense that most accurately represents space, whereas the auditory system is the sense that most accurately represents time (Bresciani & Ernst, 2007; Burr, Banks, & Morrone, 2009; Guttman, Gilroy, & Blake, 2005). Based on our results, it may be that the visual cortex offers a spatial background for remapping complex spatial auditory information and transferring the audio processing from a temporal to a spatial coordinate system. When visual input is not available this transfer may not occur, resulting in auditory maps to infer complex spatial representations based only on temporal cues. This would explain why the early occipital response in blind individuals, which is specific for the construction of spatial metric in sighted individuals, is elicited by the temporal delay of the stimulus and not by its spatial position. What is the benefit of such reorganization? Having a map that contains both spatial and temporal metric information could be useful since usually, in day‐to‐day life, objects move coherently in space and time. A possible speculation is that, when the visual network for spatial metric perception is impaired, blind individuals assume environmental stimuli to have a constant velocity, inferring space from time. This strategy could help blind people to overcome metric problems by using unimpaired temporal maps to decode spatial metrics and may facilitate their interaction with others. On the one hand, the cortical reorganization that we observe in blind individuals is adaptive as it allows them to process spatial information correctly at visual and auditory cortical levels, based on its temporal representation. However, on the other hand, the reorganization of visual and auditory cortices is maladaptive when conflicting spatial and temporal information is provided, as blind individuals are deceived by the temporal cue in the spatial evaluation, perceiving an illusory spatial position of the sound, based on its temporal coordinates. This could be the reason why sighted people did not develop this strategy: when spatial and temporal information conflicts (e.g., during accelerations and decelerations) the temporal cue is not informative for inferring space and the temporal attraction produces a misperception of the stimulus. Hence, cortical reorganization does not always seem to be adaptive, in some cases resulting in a spatial misperception that can impact on capabilities for interacting with the environment. Indeed there are real‐life situations where using temporal cues to infer space, implicitly assuming constant velocity, is potentially maladaptive. This includes all cases of accelerating or decelerating environmental objects, for example, a motorbike that suddenly increases its speed. In this example, hearing sounds closer in time does not necessarily indicate that a shorter path has been traveled. Interestingly, temporal cues also influence the activation in temporal scalp sites, suggesting that they elicit an auditory illusion of sound being perceived as delivered from a different position than the real one, based on temporal, not spatial, features. Since the visual cortex aligns neural representations of space for different sensory modalities (King, 2009; King, 2014), it could be that its retinotopic nature is sufficiently dominant to also drag the early activation of temporal scalp sites involved in the auditory processing of sounds. Based on this explanation, when the question does not involve the spatial domain, as in temporal bisection, participants instead use their auditory temporal maps to solve the task, with no need for the involvement of occipital sites. Thus, this study contributes to the debate about the role of visual recruitment following blindness, suggesting that the mere activation of the visual cortex is not necessarily functional to behavioral advantage. The sensitivity of visual and auditory areas to temporal information seems to be adaptive, as it could give blind people a strategy to build complex spatial representations, but it also has detrimental effects because conflicting spatiotemporal information exists in our multisensory world.

These findings support the cross‐sensory calibration theory (Gori, 2015; Gori et al., 2012), which suggests that calibration of the auditory system by the visual system is fundamental for the normal development of the auditory sense of space, and the reverse for time. Cross‐sensory calibration would explain why blind subjects show a specific temporal response to the spatial bisection task, demonstrating different processing for solving Euclidean metric relationships. These processes could be mediated in sighted, but not blind, people by pathways involving the superior colliculus (King, 2014; King, Hutchings, Moore, & Blakemore, 1988). The present study adds further evidence, highlighting new possible interactions during development, not only among sensory modalities but also among spatial and temporal domains. One interpretation is that metric maps contain velocity information regarding the stimulus (considering both spatial and temporal cues) and the brain assumes the constant velocity of the stimuli. The Imputed Velocity Theory (Huang & Jones, 1982) asserts that humans intuitively attribute constant velocity to a single object apparently moving through space over time. It might be that the visual system is used to calibrate complex auditory spatial representation, transferring the audio processing from a temporal to a spatial coordinate system by assuming constant velocity. The concept of velocity may represent a channel of communication between the two sensory systems. When visual information is not available, the spatial counterpart does not seem to develop and blind individuals rely only on temporal cues to infer metric spatial information.

To conclude, our data suggest that the lack of vision hampers strategies and neural circuits underlying complex spatial metrics, driving the multi‐sensory cortical network coding space based on temporal as opposed to spatial coordinates. These findings highlight new opportunities for the development of sensory substitution devices and rehabilitation technologies for blind people, where spatial and temporal cues could be simultaneously manipulated to convey richer information.

## 4 MATERIALS AND METHODS

### 4.1 Experimental design

The sample consisted of 16 blindfolded sighted subjects (mean ± *SD* age: 42 ± 16; female = 11) and 16 early blind subjects (42 ± 15; female = 11, Table S1). Sample size was decided based on a previously published study that tested spatial bisection abilities and neural correlates in healthy adults (Campus et al., 2017). A power analysis (two‐tailed *t*‐test, Cohen's *d* = 1.35, *α* = .05) indicated a minimum of 15 participants to reach a power of .85. Participants reported normal hearing and gave written informed consent. The study was approved by the ethics committee of the local health service (Comitatoetico, ASL 3, Genova) and conducted in line with the Declaration of Helsinki. Three short sounds (namely, S1, S2, S3; 500 Hz, 75 ms duration, 60 dB SPL at the subject position) were delivered at three different spatial positions and times using free‐field speakers placed in the lower visual hemifield (Figure 1b). The setup was the same as that employed in (Campus et al., 2019). The first (S1) and third sound (S3) were always delivered at −25° and +25°, respectively, with temporal separation fixed at 1,500 ms. From trial to trial, the second sound (S2) could come in space from either −4.5° (left) or 4.5° (right), and independently in time at either −250 ms or +250 ms with respect to the halfway point of the trial duration (Figure 1a). These values correspond to ~75% of correct answers for space and time bisection thresholds evaluated in a preliminary session on five subjects (Campus et al., 2019). Subjects performed two bisection tasks, one spatial and the other temporal, in two separated blocks. The order of the blocks was randomized between subjects. In each block, subjects evaluated whether the first distance/interval (between S1 and S2) was smaller or larger than the second distance/interval (between S2 and S3) in space (referred as “narrow” and “wide,” respectively), or in time (refereed as “short” and “long,” respectively). Each block consisted of 60 trials × four conditions: (a) S2 from −4.50° at −250 ms (i.e., *narrowSpace_shortTime*: first distance/interval narrow in space and short in time), (b) S2 from −4.50° at +250 ms (i.e., *narrowSpace_longTime*: first distance/interval narrow in space and long in time), (c) S2 from +4.50° at −250 ms (i.e., *wideSpace_shortTime*: first distance/interval wide in space and short in time), and (d) S2 from +4.50° at +250 ms (i.e., *wideSpace_longTime*: first distance/interval wide in space and long in time). Narrow (i.e., narrowSpace) and wide (i.e., wideSpace) first spatial distance corresponded to S2 delivered from the left (−4.5°) or right (+4.5°) side of the subject, respectively. Importantly, the stimuli were identical between the two blocks. To avoid stereotypical responses, S2 was also presented at 0° and at 0 ms during catch trials (i.e., *equalSpace_equalTime*; number of catch trials = 15). Intertrial intervals were 1,250 ± 250 ms. The temporal separation between sounds was large enough to allow a complete decay of the ERP response. To avoid possible spurious neural responses, subjects were asked to answer immediately after S3. We measured subjects' execution times (i.e., time between S3 and button press; recorded to ensure participants were focusing on the task), and performance (i.e., percentage of correct responses).

### 4.2 EEG data collection and analysis

EEG was recorded from 64 scalp electrodes using the Biosemi ActiveTwo EEG System (Figure 1c). We applied the same procedure as described in previous studies (Campus et al., 2017, 2019). Data were sampled at 512 Hz (2048 Hz with a decimation factor of 1/4) with pass‐band from DC to 134 Hz. We recorded EOG in order to discard trials showing horizontal ocular movements. EEG was filtered between 0.1 and 45 Hz. Stereotypical and nonstereotypical transient high‐amplitude artifacts were removed using Artifact Subspace Reconstruction, which is available as a plug‐in for EEGLAB software (Delorme & Makeig, 2004; Mullen et al., 2013). EEG data were further cleaned using Independent Component Analysis (Delorme & Makeig, 2004). Specifically, two EEGLAB toolboxes were used, namely, SASICA (Chaumon, Bishop, & Busch, 2015) and IC_MARC (Frølich, Andersen, & Mørup, 2015), keeping parameters as their default. Data were referenced to the average of left and right mastoids.

To test our hypothesis, namely that temporal cues alter the recruitment of the visual and auditory cortices during spatial bisection in blind individuals, we focused the analyses on the early cortical responses to S2 of the spatial bisection task, comparing blind and sighted subjects when coherent and conflicting spatiotemporal information was delivered. The responses to S2 during a temporal bisection task were used as a control to show that the conflicting spatiotemporal information was affecting the spatial but not the temporal performance and neural circuits of blind individuals.

Statistical analyses were conducted to investigate differences in the behavioral performance between sighted and blind groups, in spatial and temporal bisection. For each bisection task (*Space*, *Time*), comparisons between percentages of correct responses were performed by two‐way ANOVA, considering GROUP (*S*, *EB*) as a between‐subjects factor, and CONDITION (*narrowSpace_shortTime*, *narrowSpace_longTime*, *wideSpace_shortTime*, *wideSpace_longTime*) as a within‐subjects factor. Posthoc comparisons were conducted with two‐tailed *t*‐tests, with probabilities treated as significant when lower than .05 after Bonferroni correction.

EEG data were averaged in synchrony with S2 presentations to obtain the ERP, considering a period of 200 ms before the beginning of each trial as a baseline. We presented 60 trials for each block and condition and we required a minimum of 40 trials for each ERP, for the four spatial and four temporal conditions after artifact removal (the number of trials was ~55 per subjects). Catch trials were not considered for statistical analyses of performance and ERPs. Based on our hypothesis (Campus et al., 2019), we considered a time window between 50 and 90 ms after the sound and electrodes linked to visual (O1, O2 in occipital areas) and auditory (T7, T8 in temporal areas) processing. Mean ERP amplitude was computed by averaging the voltage in the selected time window. For each bisection task (*Space*, *Time*) and neural area (*Occipital*, *Temporal*), we performed statistical comparisons using ANOVA considering factors of HEMISPHERE (*Left*, *Right*), CONDITION (*narrowSpace_shortTime*, *narrowSpace_longTime*, *wideSpace_shortTime*, *wideSpace_longTime*), and GROUP (*S*, *EB*). Follow‐up ANOVA and posthoc two‐tailed *t*‐tests were performed, with probabilities retained as significant when lower than .05 after Bonferroni correction. For each condition of the spatial bisection task, the association between individual performance and ERP response in occipital and temporal areas was investigated using linear regression of individual mean ERP amplitude in the 50–90 ms time window, against the percentage of trials in which subjects perceived the first distance as wider. Specifically, for each condition and subject separately, we divided the number of trials in which the first distance (between S1 and S2) was reported to be wider by the total number of trials for that condition, then multiplied by 100. Such percentages were then Z‐transformed removing the group mean and dividing by the group standard deviation. This operation did not in any way alter correlations, but allowed for a better graphical representation of results by removing possible dishomogeneities in scales and/or offsets (see Figure 6).

To estimate the cortical generators of the ERP components, influenced by the experimental factors during the spatial bisection task, a distributed sources analysis was performed with Brainstorm software (Tadel, Baillet, Mosher, Pantazis, & Leahy, 2011), closely following procedures previously described (Campus et al., 2017). We averaged source activation for each subject of the two groups and condition within the selected time window after S2. Subsequently, we estimated the norm of the vectorial sum of the three orientations at each vertex. Pairwise comparisons were investigated with paired *t*‐tests, using *p* = .0001 as a threshold after FDR correction (Benjamini & Hochberg, 1995). To verify the specificity of the activation after S2 in the spatial bisection task, we compared the sighted group with the blind group, considering each condition of the task separately.

#### ACKNOWLEDGMNTS

Authors would like to thank both blind and sighted adults for their willing participation in this research.

## CONFLICT OF INTEREST

The authors declare no competing interest.

## Supporting information


**Figure S1** ERPs (mean ± *SEM*) elicited in central electrodes by S2 during space (a) and time (b) bisection task. Both left (c1) and right (c2) electrodes are reported for sighted (S) and early blind subjects (EB), considering each coherent (i.e., *NarrowSpace_shortTime*, *WideSpace_longTime*) and conflicting (i.e., *NarrowSpace_longTime*, *WideSpace_shortTime*) condition. On the *x*‐axis, *t* = 0 is sound onset. The shaded area delimits the selected time window (50–90 ms).

**Figure S2** ERPs (mean ± *SEM*) elicited by S2 during time bisection task in occipital (a) and temporal (b) electrodes. Both left (O1, T7) and right (O2, T8) electrodes are reported for sighted (S) and early blind subjects (EB), considering each coherent (i.e., *NarrowSpace_shortTime*, *WideSpace_longTime*) and conflicting (i.e., *NarrowSpace_longTime*, *WideSpace_shortTime*) condition. On the *x*‐axis, *t* = 0 is sound onset. The shaded area delimits the selected time window (50–90 ms).

**Table S1** Clinical details of early blind (EB) participants. The table shows age at test, gender, pathology, and age since subjects became completely blind.

## Data Availability

The datasets analyzed during the current study are available from the corresponding author on reasonable request.
