# Supplementary material for: Temporal cues trick the visual and auditory cortices mimicking spatial cues in blind individuals
Source: Hum Brain Mapp. 2020 Feb 12;41(8):2077–91. doi: 10.1002/hbm.24931 (PMC7267917; doi:10.1002/hbm.24931)
Supplement: Supplementary file 1 — Figure S1 ERPs (mean ± SEM) elicited in central electrodes by S2 during space (a) and time (b) bisection task. Both left (c1) and right (c2) electrodes are reported for sighted (S) and early blind subjects (EB), considering each coherent (i.e., NarrowSpace_shortTime, WideSpace_longTime) and conflicting (i.e., NarrowSpace_longTime, WideSpace_shortTime) condition. On the x‐axis, t = 0 is sound onset. The shaded area delimits the selected time window (50–90 ms). [file HBM-41-2077-s001.docx]

**Supplementary material**

**Temporal cues trick the visual and auditory cortices mimicking spatial cues in blind individuals.**

**Temporal cues trick the blind sensory cortices**

Monica Gori^1^, Maria Bianca Amadeo^1^, Claudio Campus^1^

^1^U-VIP Unit for Visually Impaired People, Fondazione Istituto Italiano di Tecnologia, Via Enrico Melen 83- 16152 Genova (Italy).

*Correspondence: [Monica.Gori@iit.it](mailto:Monica.Gori@iit.it), Fondazione Istituto Italiano di Tecnologia, Enrico Melen 83- 16152 Genova (Italy).

**
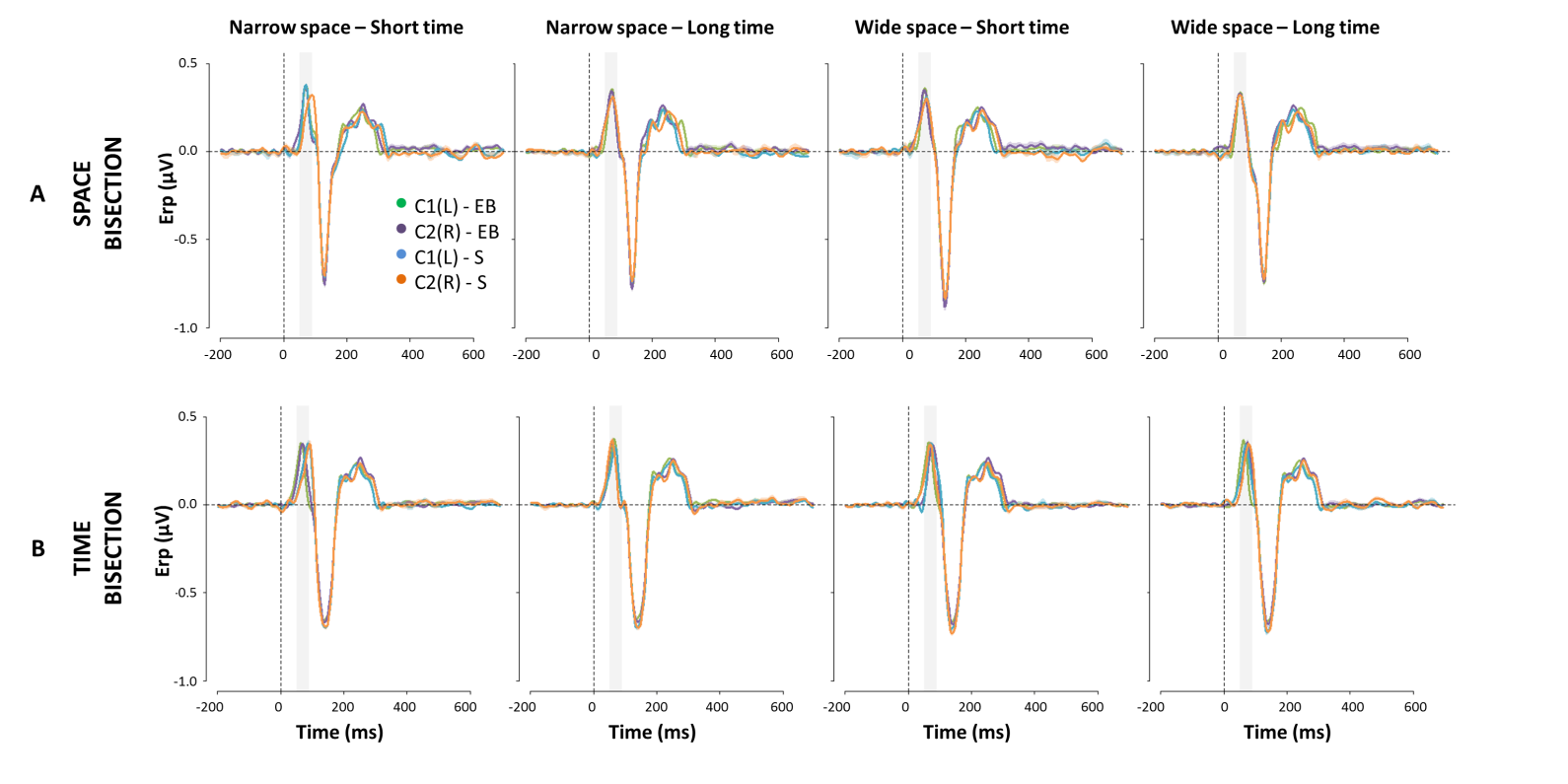
**

**Supplementary Fig.1 ERPs (mean±SEM) elicited in central electrodes by S2 during space (A) and time (B) bisection task.** Both left (C1) and right (C2) electrodes are reported for sighted (S) and early blind subjects (EB), considering each coherent (i.e. *NarrowSpace_shortTime*, *WideSpace_longTime*) and conflicting (i.e. *NarrowSpace_longTime*, *WideSpace_shortTime*) condition. On the x-axis, t=0 is sound onset. The shaded area delimits the selected time window (50–90 ms).

**
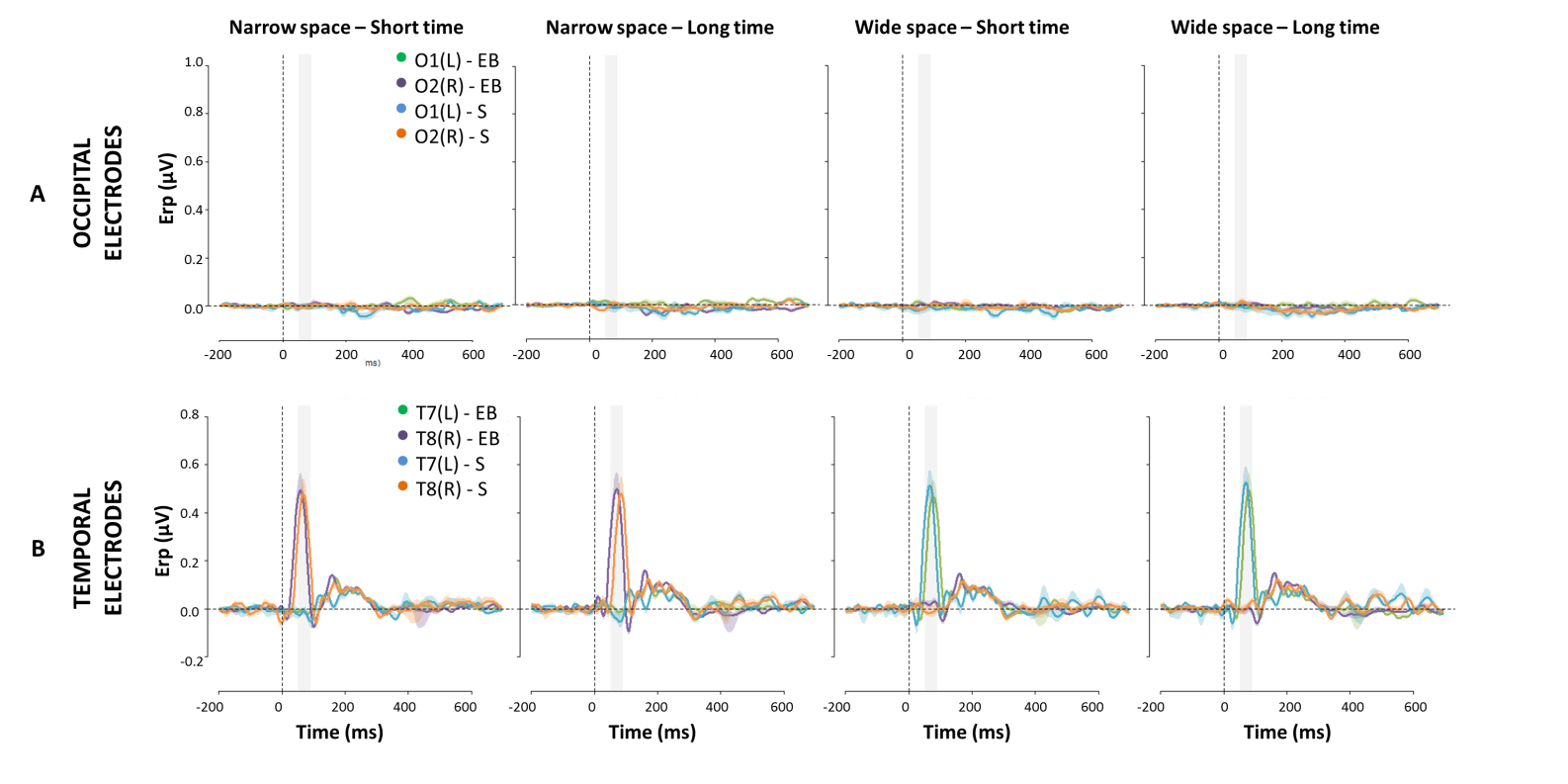
Supplementary Fig.2 ERPs (mean±SEM) elicited by S2 during time bisection task in occipital (A) and temporal (B) electrodes.** Both left (O1, T7) and right (O2, T8) electrodes are reported for sighted (S) and early blind subjects (EB), considering each coherent (i.e. *NarrowSpace_shortTime*, *WideSpace_longTime*) and conflicting (i.e. *NarrowSpace_longTime*, *WideSpace_shortTime*) condition. On the x-axis, t=0 is sound onset. The shaded area delimits the selected time window (50–90 ms).

| Participant | Age at test | Gender | Pathology | Age complete blindness |
| --- | --- | --- | --- | --- |
| EB1 | 38 | M | Retinopathy of Prematurity | Birth |
| EB2 | 25 | F | Retinopathy of Prematurity | Birth |
| EB3 | 49 | M | Retinopathy of Prematurity | Birth |
| EB4 | 20 | F | Congenital Glaucoma | Birth |
| EB5 | 72 | F | Depth damage of vision in both eyes | Birth |
| EB6 | 52 | F | Atrophy of the eyeball | Birth |
| EB7 | 38 | F | Retinopathy of Prematurity | Birth |
| EB8 | 26 | F | Retinitis pigmentosa | Birth |
| EB9 | 55 | M | Uveitis | Birth |
| EB10 | 28 | F | Retinopathy of Prematurity | Birth |
| EB11 | 22 | F | Congenital Glaucoma | Birth |
| EB12 | 60 | F | Atrophy of the eyeball | Birth |
| EB13 | 56 | M | Congenital glaucoma | Birth |
| EB14 | 38 | F | Congenital cataracts and malformation of the lens | Birth |
| EB15 | 55 | M | Retrolental fibroplasia | Birth |
| EB16 | 48 | F | Retinitis pigmentosa | Birth |

**Supplementary Table 1 Clinical details of early blind (EB) participants.** The table shows age at test, gender, pathology, and age since subjects became completely blind**.**
